# Data in ambulatory care logistics: What modelers need and what practice can offer

**DOI:** 10.1007/s10729-025-09714-w

**Published:** 2025-06-04

**Authors:** Anne Zander, Melanie Reuter-Oppermann

**Affiliations:** 1 Department of Applied Mathematics and Center for Healthcare Operations Improvement and Research, University of Twente, Enschede, The Netherlands; 2 Department of Health, Care and Public Health Research Institute (CAPHRI), Maastricht University, Maastricht, The Netherlands

**Keywords:** Ambulatory care, Healthcare systems, Healthcare planning, Data requirements, Data availability, Operations research

## Abstract

Ambulatory care facilities play a critical role in many healthcare systems worldwide. To ensure efficient care provision, we must match care demand with care supply. To support provider decision-making, this article reviews Operations Research planning problems, the corresponding planning and control decisions that must be made when opening up or running an ambulatory care facility, and their data requirements. We give an overview of demand and supply-related data that an ambulatory care facility can collect and comment on the consequences for decision-making if some of that data is missing. We briefly discuss three healthcare systems and their influence on data collection and decision-making. We also take a closer look at several real-world appointment data sets and their usefulness for planning decisions. In addition, we discuss model implementation barriers and give recommendations for modelers and practitioners to bridge the gap between theory and practice. Finally, we present future research directions for Operations Research in ambulatory care.

## Highlights

 

- We review planning and control decisions for ambulatory care facilities with a special focus on data requirements.
- We describe the attributes of a comprehensive supply and demand data set that an ambulatory care facility can collect.
- We comment on the consequences for decision-making if certain data is unavailable.
- We discuss three healthcare systems and their influences on data collection and decision-making.
- We illustrate barriers to the implementation of Operations Research models and propose solutions.

## Introduction

In many countries, ambulatory care facilities, called practices in the following, are responsible for delivering the majority of primary care and even some secondary and tertiary care [42]. Hence, a considerable lever to improve care is to support provider decision-making for matching supply and demand for their practice. On the one hand, this benefits patients, resulting in better access to care and less waiting time. On the other hand, it helps providers establish a reliable working environment, avoiding financial loss and extensive overtime, making the medical profession more attractive.

Even though healthcare systems worldwide differ significantly, in most countries, practices need sufficient patients to be profitable and attractive for physicians to work in. At the same time, ambulatory care, especially primary care, is crucial for people’s welfare. Patients benefit from having easy and timely access to care nearby. Most Western countries face a shortage of physicians, including general practitioners, especially in rural areas [40, 59, 65, 77, 120]. One cause is the ageing population that needs more medical care, and another is the shrinking working population [93].

Operations Research (OR) and Operations Management (OM) approaches have been successfully developed for and applied to many different healthcare problems [19, 50], and they also offer great potential to support ambulatory care logistics. The main goal of this article is to provide insights into the connection between planning problems considered in Operations Research and their need for data, especially the data that can be collected by an individual practice. To this end, we review planning problems and their corresponding planning and control decisions, assign them to the three main planning levels: strategic (long-term), tactical (mid-term), and operational (short-term), and report on their data requirements. We present a best-case practice data set that consists of all supply and demand data that a practice can collect, and comment on the consequences for decision-making if some of this data is missing.

Laws and regulations can often influence planning problems, decision-making, and data collection in ambulatory care logistics. We discuss examples based on the German, Dutch, and New Zealand healthcare systems. These three countries were chosen as examples because in all three countries, health coverage is guaranteed for everyone in the Commonwealth, in contrast to the US, for example, but there are still some differences between the healthcare systems. While in the German and Dutch healthcare systems, compulsory health insurance pays for patients’ care, in New Zealand, hospital care is tax-funded, but patients often need to pay at least part of the costs for primary care themselves. In Germany, patients can freely choose a general practitioner (GP), and only very few ask patients to register with them, but GPs can also decline to add new patients to their panel [27, 103]. In the Netherlands, registration with a GP is mandatory in order to get an appointment, and while patients can freely choose a GP, they might decline the patient’s wish to register [101, 104]. In New Zealand, patients can freely choose the GP (but not the hospital), and if they register with a GP, their appointments and treatments are subsidised by the government [72, 105].

Out of the three countries, Germany spends the most on healthcare, with 13% of GDP. The Netherlands and New Zealand spend around 11% and 10% [36]. In comparison, the US spends nearly 18% of its GDP on healthcare [36].

Further, based on real-world appointment data sets from different German practices, we show which data is available and which is missing and discuss whether or not decisions can be supported. Finally, we comment on barriers to applying Operations Research models in practice and propose solutions to eliminate those barriers and future research directions.

This article is organized as follows. Section 2 reviews the planning problems and lists the planning and control decisions, including data requirements, that need to be taken by the practice, sorted by planning level. In Section 3, we present a best-case practice data set. Next, we comment on the importance of collecting specific data in Section 4. We discuss data availability and specifics for the three countries of Germany, the Netherlands, and New Zealand in Section 5. Besides, we analyse several real-world data sets and compare them to the best-case practice data set. Then, we link our findings and describe barriers to applying Operations Research models in practice in Section 6. Last but not least, in Section 7, we draw a conclusion and give an outlook on possible further research.

**Table 1:** Regional strategic planning

Planning problems and decisions	Objectives	Input parameters	Data
Service design, location, and capacity planning Determine types, numbers, locations, and capacities (numbers of physicians in FTEs) of medical practices [43]	Minimize cost/number of practices or physicians [43] Minimize the access cost for patients (e.g., travel cost, travel distance, or travel time, or technology costs) [34] Maximize covered demand [34] Maximize equity in access [34]	Number of new practices and physician full time equivalents (FTEs) to place or minimal service level, e.g., w.r.t. coverage [64] Projected types, numbers, locations, and capacities of existing medical practices over time [99] Panel size per physician FTEs and practice type: Patient number/demand volume that can be serviced by a single physician [64] Set of potential practice locations (incl. Internet availability) [86] Set of patient locations (incl. Internet availability and affinity ) [86] Projected demand volume per patient location and practice type over time Projected patient choice behavior Access cost (travel time, distance, or distance) between potential practice locations and patient locations per mobility mode [64] or technology costs (e-visits)	Number of new practices and physician FTEs to place or minimal service level, e.g., w.r.t. coverage [64] Numbers and types of physicians in training expected to work in the region Demography of practicing physicians in the region [64] Set of potential practice locations (incl. internet availability) [86] Demographic and socioeconomic data on the regional patient population [64] Spatial distribution of regional patient population [64] Mobility of the regional patient population and mobility services offered in the region Technology commitment, Internet availability (in catchment area) Historical demand of the regional patient population (and other similar patient populations) per practice type

**Table 2:** Strategic planning part 1

Planning problems and decisions	Objectives	Input parameters	Data
Service design and case-mix			
planning: Target patient types Offered medical services (this includes, e.g., e-visits and home visits) [43]	Maximize profit	Profit per potential service and patient type	Allowed service designs and case-mixes based on higher-ranking regional decisions Compensation and costs of services per patient type (includes e-visit costs, e.g., software, headsets, camera, and telehealth costs) [13]
Location planning: Practice locations [43]	Maximize profit Minimize access cost for patients [34] Maximize covered demand/access [34] Maximize equity in access [34]	(Projected) number, locations, and capacities of existing practices that offer similar services Set of potential practice locations and their fixed and variable costs Set of patient locations Demand volume and profit per patient location and chosen practice locations Access cost (travel cost, time or distance) between potential practice locations and patient locations per mobility mode	Service design and case-mix decisions Number, locations, and capacities of existing practices that offer similar services Set of potential practice locations and their fixed and variable costs as well as their features such as Internet connection (potentially based on higher-ranking regional decisions) Demographic and socioeconomic data on the regional patient population Spatial distribution of the regional patient population Mobility of the regional patient population and offered mobility services Historical demand of the regional patient population (or other similar patient populations)

**Table 3:** Strategic planning part 2

Planning problems and decisions	Objectives	Input parameters	Data
Capacity planning: Number and types of rooms [43] Number and types of (expensive) equipment [43] Opening/consultation hours [43] Workforce planning: *Staffing:* Number and types of physicians and non-physician staff (including working hours per staff member) [43]	Maximize costs [25] Maximize access [43]	Projected demand volume for services Staff and resource (i.e., rooms and equipment) requirements per service Service durations	Allowed capacity based on higher-ranking regional decisions Service design, case-mix, and location decisions Historical demand volume per service of the regional patient population (or other similar patient populations) Staff and resource (i.e., rooms and equipment) requirements per service, including corresponding adjacent, e.g., admirative, staff and resource requirements Historical service durations
Layout planning: Sizes and locations of rooms [5] Assignment of functionalities to rooms	Minimize walking distances for physicians, non-physician staff, and patients [5]	Number and min. sizes of rooms (per functionality) [5] Potentially maximum floor plan size or outer walls [5] Walking pathways between (functional) rooms of physicians, non-physician staff, and patients and their frequencies [5] Legal and regulatory constraints Preference and special requirement (soft) constraints	Service design, case-mix, location, capacity and staffing decisions Care pathways [5] Historical demand volume per care pathway of the regional patient population (or other similar patient populations) Planned working processes of care pathways, including patient, physician, and non-physician walking pathways between (functional) rooms [5] Laws and guidelines Special requirements and preferences

**Table 4:** Tactical planning part 1

Planning problems and decision	Objectives	Input parameters	Data
Panel management: Patient types that will be accepted or rejected to enter the practice or the physician panels [119] Workforce and capacity planning: Adjust staffing and opening/consultation hours [43] Manage staff (hire, lay off, and adjust working contracts) [22] Back-up physician(s) per panel in case of absence of the original physician	Minimize deviation between the projected workload generated by the panels and the available capacity over time [119]/overflow frequency [74] Maximize continuity of care [74] Maximize fairness between physicians [119] Maximize profit	Current panel compositions, i.e., number of patients per type [119] Projected future demand per patient type to enter the panel(s) [119] Average workload generated by one patient of a certain type for physicians, non-physician staff, and resources [119] Transition probabilities between patient types over time [119] Profit per patient type Projected availability and capability of staff	Service design, case-mix, location, staffing, opening/consultation hours decisions Currently employed physicians and their working contracts [119] Current panel patients of the practice [119] Historical demand and supply data from the practice or similar practices [119] Compensation and costs of services per patient type Staff composition Projected availability and capability of staff
Workforce planning: *Shift design and physician* *scheduling:* Length and start time of shifts [26] Number and types of non-physician staff per shift [26] Working days and times of physicians [25] *Capacity allocation:* Services per shift, including assignment of resources Services per physician and time interval, including assignment of resources	Minimize set-up costs and times Maximize physician satisfaction	Constraints based on labor law regulations, staffing, and physician contracts Preferences of physicians Projected demand volume for services per staff member type and physician and their timely relation due to the care pathways Service durations Resource (i.e., rooms and equipment) requirements per service	Service design, and staffing decisions Care pathways and their implementation in processes Historical demand volume per care pathway of the regional patient population (or other similar patient populations) Labor law regulations Physicians with their panels and working contracts Preferences of physicians
Appointment planning: *Admission control* *and access policy:* Allowed patient types, care pathway, request mode, request time, and urgency level combinations per service, time interval, staff member/physician, and resource combination [43] Rules for handling cancellations and no-shows, e.g., allow rescheduling or charge fees [3] Rules for handling walk-ins, urgent patients, unpunctual patients [43]	Minimize rejections [23] Minimize access time/indirect waiting time [43] Minimize violations of access time targets [3] Maximize fairness between patient types [3] and care pathways Minimize no-shows and cancellations [58]	Access time targets/priorities [78] Projected distribution of service requests per patient type, care pathway, request mode, request time, urgency level, service, and requested time of service and physician combination [23] Service durations per service (pot. also per patient type, care pathway, type staff member/physician, etc.) [89] Cancellation and no-show probability per patient type, care pathway, request mode, request time, urgency level, service, type staff member/physician and time of service combination [89]	Panel management, shift design, physician scheduling, capacity allocation decisions Care pathways Historical demand and supply data from the practice or similar practices [56]

**Table 5:** Tactical planning part 2

Planning problems and decisions	Objectives	Input parameters	Data
Appointment planning: *Appointment scheduling:* Create blueprints: Define service start times (multiples of time slots) reserved for certain services, patient types, care pathways, request modes, request times, and urgency levels Rules to decide which patient in the waiting room to see next [43]	Minimize access time/indirect waiting time [14] Minimize violations of access time targets [60] Minimize idle time [43] Minimize overtime [43] Minimize direct waiting time [43]	Access time targets/priorities [78] Projected distribution of service requests per patient type, care pathway, request mode, request time, urgency level, service, and requested time of service and physician combination and their timely relations due to the care pathways [113] Distribution of service durations per service (pot. also per patient type, care pathway, type staff member/physician, etc.) [52] Cancellation and no-show probability per patient type, care pathway, request mode, request time, urgency level, service, type staff member/physician and time of service combination [20] Punctuality distributions per patient type, care pathway, request mode, urgency level, service, staff member/physician and time slot combination [113]	Panel management, admission control and access policy decisions Care pathways Historical demand and supply data from the practice or similar practices [56]
Workforce planning *Rostering:* Non-physician staff is assigned to the predefined shifts [11, 43] Adjustment of physician schedules (and consequently, admission control and appointment scheduling) based on planned absences of physicians [25]	Minimize costs [11] Maximize fairness [11] Maximize satisfaction [11] Maximize robustness	Shift design [11] Number and types of staff [11] Constraints based on labor law regulations, working contracts and previous rosters [11] Preferences/availabilities of staff [11] Back-up physicians	Shift design decisions Staff composition Labor law regulations Working contracts Previous rostersPreferences/availabilities of staff Back-up physicians

**Table 6:** Operational planning

Planning problems			
and decisions	Objectives	Input parameters	Data
Panel management: Accepting or rejecting requests to enter a panel [119] *Patient-to-panel* *assignment:* In case of acceptance, assign the patient to a physician panel [119]	Maximize fulfilment of patient preferences Maximize fairness between physicians [119]	Rules from capacity and panel management Requesting patient type [119] and pot. physician preferences	Panel management decisions Current panel compositions [119] Individual features of requesting patient [119] Physician preferences of requesting patient
Appointment planning: *Handling appointment* *requests:* Accepting, rejecting, or re-directing requests for an appointment (may include requests for service, physician, time slot) or several appointments along a care pathway [3] *Patient-to-appointment* *assignment:* In case of acceptance, assign appointment requests to services, physicians, and time slots (start times) [14, 43]	Minimize access time/indirect waiting time [3] Minimize idle time [14] Minimize overtime [14] Minimize direct waiting time [14] Maximize fulfilment of preferences of patients [3] Minimize deviation from the care pathway	Rules from admission control/access policy, appointment scheduling Current schedule Individual features of requesting patient (may include preferences) [29] Projected future appointment (and walk-in) demand [29]	Admission control, access policy, and appointment scheduling decisions Current schedule Individual features of requesting patient (may include preferences) Historical demand and supply data from the practice or similar practices
*Handling walk-in* *requests:* Accepting, rejecting, or re-directing requests to walk in (may include requests for service and physician) [83] Assign accepted walk-ins to a physician	Minimize overtime [83] Minimize idle time [83] Minimize rejections [83] Minimize direct waiting time [83] Maximize fulfilment of preferences of patients	Appointment scheduling rules Today’s schedule Number and waiting time of currently waiting patients per request mode, service, and staff member type/physician [83] Requesting patient type Projected future demand for today	Appointment scheduling decisions Today’s schedule Number and waiting time of currently waiting patients per request mode, service, and staff member type/physician Individual features of requesting patient Historical demand and supply data from the practice or similar practices
*Queue discipline:* Whom to serve next [43]	Minimize idle time [90] Minimize direct waiting time [90]	Appointment scheduling rules Number and waiting time of currently waiting patients per urgency level, service, and staff member type/physician [91] Projected future patient arrivals per urgency level, service, and staff member type/physician [91]	Appointment scheduling decisions Number and waiting time of currently waiting patients per urgency level, service, and staff member type/physician Historical demand and supply data from the practice or similar practices
Appointment and workforce planning: *Dynamic patient (re)-assignment/staff* *rescheduling:*Adjust roster, schedule, and waiting list (patients waiting in the waiting room) to react to a deviation between plan and realization [43]	Minimize access time/indirect waiting time [43] Minimize overtime [43] Minimize idle time [43] Minimize rejections [43] Minimize direct waiting time [43]	Higher-level rules [43] Current schedule, roster, waiting list [43] Changes in planned demand or supply [43]	Higher-level rules Current schedule, roster, waiting list Current disruptive event

## Planning problems and decisions

In this section, we review the planning problems and the corresponding planning and control decisions that need to be taken by the practice management on the different planning levels: strategic (long-term), tactical (mid-term), and operational (short-term), and relate them to the data needed for the considered decisions. For completeness, we also include strategic decisions that fall within the responsibility of some higher regional authority. The mapping of planning and control decisions to planning levels and the naming mainly follow Hulshof et al. [43]. For each planning level, we present tables with four columns, where the first column indicates the planning problem, potential subproblems, and the corresponding decisions on the considered planning level. The second column lists the corresponding objectives, the third the required input parameters, and the fourth shows the data to define those model input parameters. Tables 1, 2 and 3 address the strategic level, while Tables 4, 5 target the tactical and Table 6 the operational planning level. Here, when possible, we include at least one reference per entry in the tables. Note that the references do not always exactly state the elements that we list in the table, but parts of them or at least go in the same direction. This is because we include already proposed input parameters and those that we think are reasonable. We will comment on some of those in relation to future research in Section 7.

Note that considering several objectives simultaneously when planning usually leads to trade-offs. For example, in the case of appointment scheduling in Table 5, the objectives of idle time and overtime are conflicting. Here, methods from Operations Research for multi-objective problems can be employed to find acceptable solutions.

Only a few papers work with real-world data, which explains the missing references in column 4. However, the necessary data can often be reconstructed from column 3 on input parameters, since input parameters are often predicted values based on historical data.

**Fig. 1:**
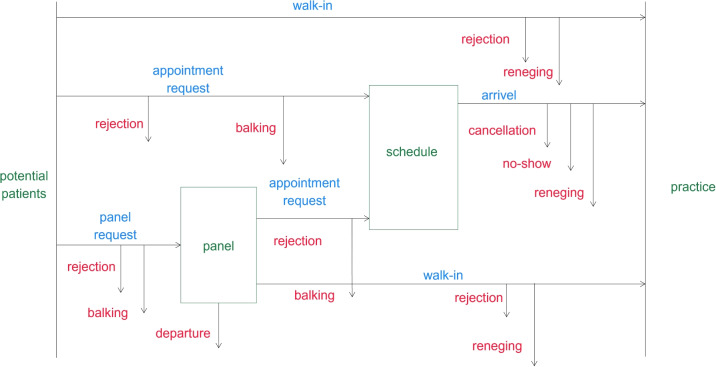
Possible patient demand behavior

Concerning the input parameters and data columns, we report them on the most detailed level that is appropriate to address the decisions. However, note that sometimes decisions can be made based on aggregated versions of entries in columns 3 and 4. When using the term historical demand and supply data in column 4, we refer to the data attributes described in Section 3, which illustrates what data can or should be collected by individual practices. For the demand part, the data attributes correspond to data collection on patient-practice interactions, as illustrated in Fig. 1. In the figure, balking refers to a patient who observes the current system state and decides not to seek service after all. In contrast, reneging refers to a patient who starts waiting for service but then decides to abort before being served.

For the supply part, the data attributes refer to documenting supply decisions. Note that the historical patient demand is also shaped by the then-implemented planning and control decisions and external factors, e.g., the season or the weather. Hence, it is recommended that this data be collected as well. In some cases, data might not be available, and proxy data can be used, or assumptions have to be made. For example, when opening a new practice, historical data of that practice has not yet been collected. Then, historical data from other practices or publicly available data (on patient demographics) can be used. Another example regarding appointment scheduling would be that the realized appointment durations are not documented. Then, estimates, e.g., by the physician, regarding the appointment duration distribution can be used instead.

Further, there are decisions that, in general, will not be optimized but will just be taken based on practical reasons, such as the time unit considered to measure appointment slot lengths. We also considered those types of decisions as data.

In general, making decisions in an integrated way will lead to better results. However, due to complexity, decisions are often made separately. In that case, decisions on higher planning levels guide decisions on lower ones. They can, therefore, also be seen as input data or input parameters to those lower-level decisions.

**Fig. 2:**
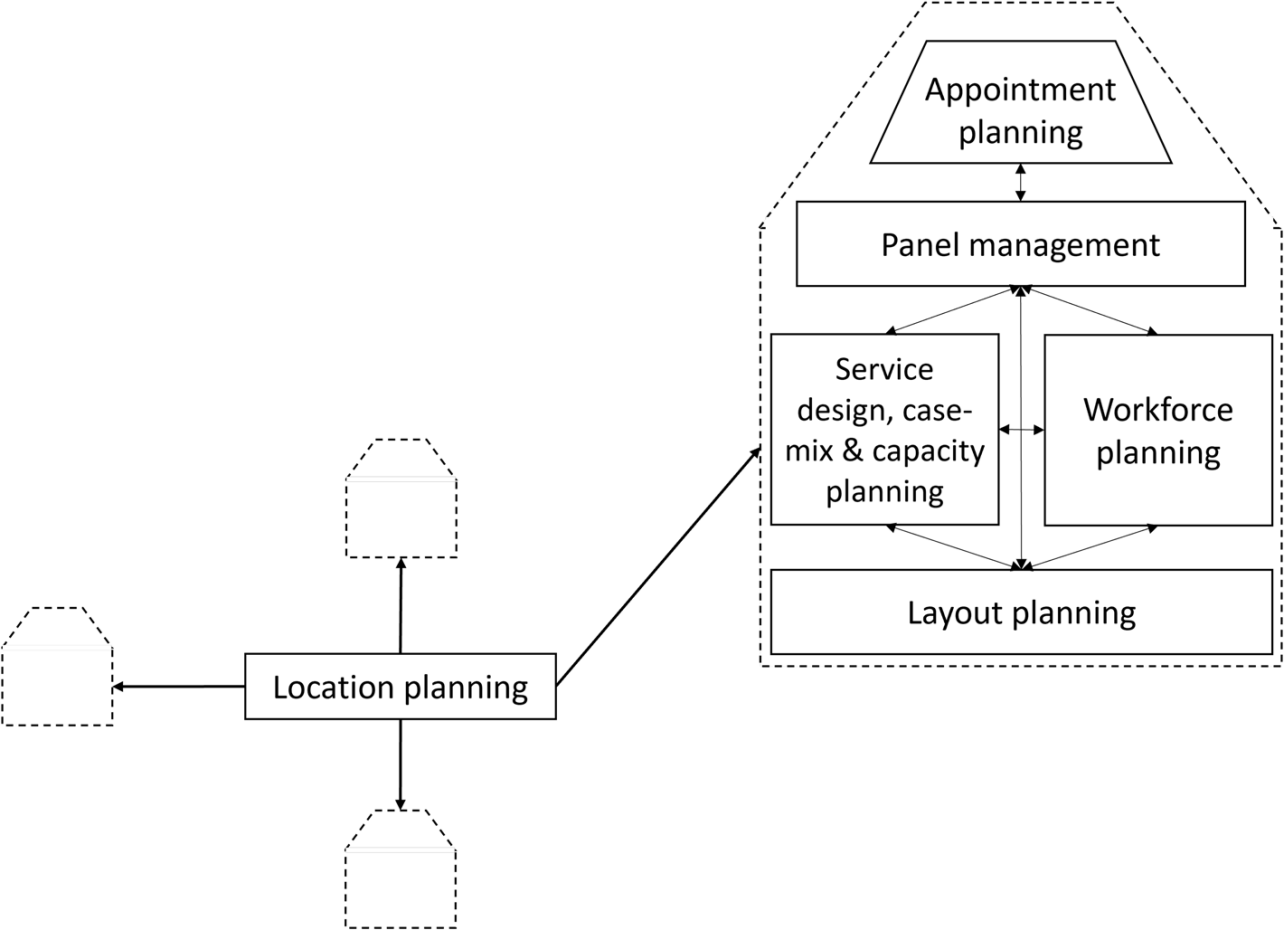
Illustration of planning problems for ambulatory care practices

In the following, we perform a qualitative literature review, where we list a few relevant, recent, exemplary references for each of the considered planning problems in our tables: Location planning, layout planning, service design planning, case-mix planning, capacity planning, workforce planning, panel management, and appointment planning. The considered planning problems are also displayed in Fig. 2. In addition, we added a section related to different types of access channels, with a focus on e-visits. In our literature review, we will comment on the types of models and solution approaches used to tackle the planning problems and decisions listed in the tables. Further, we will give more details on the necessary data collection, where necessary.

We begin with reviewing articles that consider relevant planning problems as well as corresponding planning and control decisions. Hulshof et al. [43] present a taxonomic classification of planning decisions in health care, with one part of the article explicitly targeting ambulatory care services. Rais and Viana [82] present a survey on Operations Research applied to healthcare. Jack and Powers [45] review articles on demand management, capacity management, and performance in health care. Zonderland and Boucherie [125] review patient planning and scheduling, proposing a framework considering planning/hierarchical levels, services, and the planning complexity. Further, Zonderland [124] reviews outpatient clinic optimization. While those outpatient clinics are part of a hospital, there is much overlap with optimization problems for independent medical practices. Finally, Youn et al. [112] review research on planning and scheduling in healthcare.

### Location planning

Location planning has been widely studied within the OR literature in general [24] and also for many healthcare applications like locating ambulances [84], for example. Overviews on healthcare facility location planning can be found in Ahmadi-Javid et al. [4], Daskin and Dean [21], and Güneş and Nickel [34], for example. Still, fewer publications have addressed the location planning of (primary care) practices. One reason could be that GP practices are managed individually in many countries, and their locations cannot be coordinated as easily as with other health services. In Germany, for example, decisions about the number of practices per region are made centrally, while GPs can then decide about the specific location, i.e., address, of their practice. In New Zealand, GPs can make their decision more freely. Nevertheless, as GPs are a scarce resource in many countries worldwide, it becomes increasingly crucial to place them at efficient locations and inform decision-makers from the healthcare systems and GPs about existing approaches and the possibility of receiving decision support.

In Tables 1 and 2, we summarize the objectives, input parameters, and necessary data for location planning on a regional level and on the strategic level targeted by an individual practice.

Depending on the type of facility and the healthcare system, the importance of the different objectives for the individual problem can vary. Güneş and Nickel [34] review healthcare facility location models and define the following objectives that also apply to location planning for ambulatory care practices:

1. Minimize the access cost for patients (e.g., travel cost, distance, or travel time),
2. Maximize covered demand, and
3. Maximize equity in access.

In the case of preventive care facilities, for example, visits are, in general, rare events, and often, facilities only address certain patient types [32]. On the contrary, many patients might see their GPs regularly, and equity of access and low travel costs are both very important. Then, multi-criteria approaches are of high importance, as reviewed, for example, by Farahani et al. [28].

The problem of locating primary care facilities is addressed, for example, in Abernathy and Hershey [1], Ahmadi-Javid and Ramshe [2], Graber-Naidich et al. [30], Güneş et al. [35], Hillsman [39], Hodgson et al. [41], Lopane et al. [64], Mitropoulosa et al. [75], Parker and Srinivasan [76], Reuter-Oppermann et al. [86], and Tien and El-Tell [102] with varying objectives and constraints. Besides the locations of practices, some publications also address the size of the practices, i.e., the number of physicians working in each practice, showing a connection with the workforce planning problem of staffing. To make the approaches useful for practice, they should be integrated into a decision support tool combined with a geographic information system [85]. Reuter-Oppermann et al. [86], for example, located GP practices in a German district using three model variations. These models were developed under two main basic requirements: (1) one practice is to be located that can be reached by as many inhabitants as possible, and (2) locate practices to cut down the driving time for all inhabitants to the next practice location to less than 15 minutes. The input data the authors used in their work included the demand (population), driving times, and the current GP locations. Lopane et al. [64] study access to primary care for patients in Northland, New Zealand, and propose a multi-criteria heuristic to locate GP practices.

The designs of primary care services and the underlying healthcare system significantly influence the location planning problem to be solved. The central aspect is whether patients can freely choose their practice or if they are assigned to one based on their home address. Suppose they can freely choose, then modeling their behavior, i.e., when and where they see a general practitioner, is very challenging. Panel information from existing practices would be a valuable input for location planning, but it is usually very difficult to obtain. Therefore, most publications make assumptions based on distance, for example. Others try to ensure spatial accessibility of practices for all inhabitants, as, for example, done by Schuurman et al. [94]. A review of concepts, methods, and challenges for spatial accessibility of primary care was published by Guagliardo [33], for example.

If inhabitants are assigned to practices, the location problem is a districting problem of healthcare regions. Yanık and Bozkaya [111] present a review of districting problems in healthcare with dedicated districting models for primary care. The idea is that all patients living within a district are served together. If primary care is centralized and districts are small enough, patients are served by one practice per region, and the districting problem is equivalent to a simple location allocation problem.

Especially in rural areas or developing countries with a significant GP shortage and potentially low population density but long distances between villages, mobile practices can be a good option to provide primary care to the inhabitants living in these regions. Then, the location problem is combined with a routing problem, as, for example, proposed by Hodgson et al. [41]. The strategic problem of defining which locations to visit and when by mobile practices was studied by Büsing et al. [12].

Important input parameters for location planning problems include the set of potential locations as well as representatives for patient locations. In addition, a distance matrix between the two location sets must be computed. Assumptions on how and where patients attend a practice must be made. A standard assumption is that patients travel by car and choose the closest practice, while a maximum driving time of, e.g., 15 minutes is targeted [86].

Most of the publications address the location planning problem on the regional strategic problem (Table 1). Therefore, only a few references exist for the strategic problem targeting only one new practice (Table 2), while most could be easily transferred and adapted.

### Layout planning

A patient’s care pathway contains multiple services, here, at the same ambulatory care practice during a patient’s practice visit. It includes several process steps and potential services connecting multiple resources that must be available. This includes registration of a patient, waiting, a doctor’s consultation, and potentially lab tests. When opening a new practice, it must be decided in which rooms the services and process steps are supposed to take place and which resources will be assigned. For example, registration is usually done by a nurse at a counter in the entrance area. Consequently, the size and order of the rooms must be decided.

In Germany, for example, many single-handed practices are built into standard apartments within apartment buildings, as one reception, one waiting room, and one or two treatment rooms are sufficient to provide the desired level of care for their patients. This might be why the literature on layout planning of practices is scarce. The practice layout becomes more important when group practices are installed with several physicians and nurses who can treat more patients simultaneously.

Several input parameters are important for the layout planning of ambulatory care practices, as summarized in Table 3. First, the number and the minimum sizes of the rooms (per functionality), sometimes with a maximum floor plan size or outer walls, are necessary inputs. This includes if one doctor uses one treatment room to treat patients one after another, or two or more, and moves between the rooms. In general, the aim is to minimize walking distances for physicians, nurses, and maybe even patients. To model that, pathways are needed as input, together with their frequencies. This can be challenging if it is an entirely new practice. Then, processes should be defined first. Staff and patient numbers should be known or possibly estimated. If physicians use two rooms, many prefer to have a door between them to switch easily. This would then constrain the order of rooms. Many additional constraints might need to be considered. For example, in primary care practices, physicians might not want to be seen by patients who pick up a prescription to avoid scheduling delays.

Once a layout is computed, a discrete-event simulation can efficiently evaluate the expected walking distances and other indicators.

Within the healthcare literature, layout planning has been applied to the design of hospitals or hospital departments. Helber et al. [38] specifically target large and complex hospitals within their research. From the operations management viewpoint, Vos et al. [109] propose a framework to evaluate hospital designs. Several publications have addressed specific hospital departments, e.g., outpatient clinics [106] or emergency departments [88]. Arnolds and Nickel [5] targeted the layout planning of hospital wards considering multiple periods.

Literature on hospital layout planning has been summarized by Jamali et al. [46]. A review on layout planning in healthcare was presented by Benitez et al. [9] and Arnolds and Nickel [6], for example, and on facility layout planning in general by Pérez-Gosende et al. [79].

### Service design, case-mix, and capacity planning

Before opening a new practice, the practice manager must decide on the services offered and the corresponding target patient groups. We have only found one publication that addresses these problems for medical practices. One reason might be the difficulty of obtaining a helpful data basis to make those decisions. Another reason may be that practices are often opened by a single physician or a group of physicians, and their specialties usually define the services that can be offered. In Germany, the specialties and the fact that the physicians are registered with statutory health insurance (or only with private health insurance companies) mostly define the target patient groups. In New Zealand, for example, an important factor for patients in choosing a practice is the prices charged for a visit.

To the best of our knowledge, only Comis et al. [18] have developed a simulation framework that allows us to model patient behavior and analyse primary care practices’ service designs.

In some countries, health departments or similar legal structures decide what types of practices can be opened or taken over. In Germany, this is the Association of Statutory Health Insurance Physicians, for example, Kassenärztliche Bundesvereinigung [National Association of Statutory Health Insurance Physicians] [49]. Still, practices can decide whether to offer extra care services or dedicated office hours for specific patients or care types.

Service design has a strong interdependence with the practice layout, staff planning, appointment planning, and panel management. Based on the services and care types offered, matching patients can be accepted to the panel until the maximum panel size is reached. This directly relates to the number of physicians and nurses working in the practice, as well as the opening hours and consultation times. The number of treatment rooms, the equipment, and the waiting room capacity should be planned accordingly.

Important input for service design planning is the (expected) demand for services, e.g., based on the inhabitants and existing practices in the catchment area. In addition, the interdependence with the other planning problems must be considered, e.g., regarding opening hours and the number of staff.

We summarize the findings on objectives, input parameters, and data in Tables 1, 2, and 3.

### Workforce planning

If a practice is not owned by the physicians but by health providers, physicians are usually employed. Then, the number of physicians to be hired must be determined, and shifts and the number of physicians must be defined to match the consultation hours. The same holds for medical assistants or nurses.

Overall, the following workforce planning problems have to be considered:

- Staffing - number of physicians, number of medical assistants or nurses,
- Shift design - number, length, and distribution of shifts within the week,
- Rostering - assignment of shifts to staff,
- Re-planning - re-assignment due to disruptions, e.g., staff sickness.

All planning problems have been addressed in the literature to a great extent for hospital nurses and physicians. In contrast, publications on ambulatory care workforce planning are scarce. We summarize the key facts on workforce planning at the strategic level in Table 3, on the tactical level in Tables 4 and 5, and on the operational level in Table 6.

Many review papers have been published, including reviews on nurse rostering [11, 16], on physician scheduling [25], on general staff scheduling and rostering [26], as well as workforce planning [22]. Stiglic and Kokol [98] propose an approach for nurse scheduling in ambulatory care centers that provide primary care for patients in Slovenia, and Nguyen et al. [73] present a stochastic linear optimization model to determine the required number of physicians for an outpatient system with patient reentry.

### Panel management

Many office-based physicians have a so-called panel, i.e., patients who regularly visit the physician. A physician-centered panel ensures continuity of care for their panel patients. However, we can also consider panels that are served by several physicians together. If a big part of the demand comes from panel patients, it is essential to manage the panel to better control and forecast demand. How can we measure the workload produced by a panel? First, we must define who belongs to the panel and who does not. Next, we need to measure the workload produced by single panel patients, i.e., the number of visits, total time spent by the physician (and non-physician staff) to care, and organize the patient’s care during a fixed period. Based on this historical data, we try to forecast the future panel demand and use this information to determine whether to accept or reject patient requests to enter the panel.

The most straightforward approach to measure the panel workload is to determine the panel size, i.e., counting the number of panel patients. However, there is no general standard on which patients to count. Some consider the patients seen in the last two years [66, 67]; others the number of patients seen in the last 18 months [70, 71, 81]. Given a time frame, we determine the average workload per panel patient. Then, the question is how to determine the maximal panel size given a physician’s capacity. Murray et al. [71] propose dividing the physician’s capacity in a given period by the average time required for a single panel patient. However, this approach completely ignores uncertainty in the problem structure.

Several queuing models have been suggested to account for stochastic demand and sometimes stochastic service times. Green and Savin [31] propose two queuing models to determine the relationship between the panel size and the expected appointment backlog, i.e., the expected number of patients with a scheduled appointment waiting to be treated. In their model, they take into account that patients do not show up for their appointments (so-called no-shows) and that those patients may reschedule. Zander [117] extends their approach by including an appointment request rate of panel patients that is dependent on the length of the appointment backlog. Liu and Ziya [61] also present two queuing models and decide on the panel size and the service capacity to maximize the long-term average reward while constraining the expected access time, i.e., the time between an appointment request and the actual service. Izady [44] uses several discrete-time queuing models with bulk service, i.e., several patients are served at the same time. In contrast, Zacharias and Armony [113] consider the appointment backlog together with direct waiting time and also decide on the panel size and the service capacity to maximize the long-term average reward. Finally, Vanberkel et al. [107] use a queuing network of multi-server queues to define the panel size of an oncology practice that balances demand from new and relapsed patients. The following information is necessary to apply the queuing models: Panel size, appointment request rate, daily appointment capacity, service time (distribution), no-show probability (possibly dependent on the indirect waiting time), rescheduling probability of no-shows, and rewards and costs.

Patients have very different medical needs. Therefore, it is reasonable to classify panel patients with similar attributes and needs instead of counting them. Balasubramanian et al. [7] propose a stochastic linear program to reassign patients to primary care physicians of a group practice with the objectives of minimizing access time and improving continuity of care. Here, they use a patient classification based on age and gender. Ozen and Balasubramanian [74] minimize the maximal probability that the daily demand exceeds capacity in a group practice of primary care physicians to redesign physician panels. Finally, Zander et al. [119] consider patient classification and consider the panel’s future evolution. They propose deterministic integer linear programs that decide on the intake of new patients into panels with the primary objective of minimizing the deviation between the expected panel workload and the physician’s capacity over time. Usually, the decisions on which patient types will be accepted to the panel are made on the tactical planning level; see Table 4. Those decisions are then implemented on the operational (online) planning level; see Table 6.

The most important historical demand and supply data needed on the tactical level is given by individual (panel) patient data such as age, gender, request time to enter a panel, the number of visits, and the time spent with different resources and the assigned panel physician(s) (if any). From there, we can classify patients and define the following input parameters: average workload per resource and single patient of a particular type, the probability to change types over time,e and the predicted future patient demand to enter a panel. On the operational level, we need to know the individual features of the patient who requests to enter the panel along the lines of our defined patient types.

### Appointment planning

In this section, we review the literature on planning decisions that relate to the handling of appointments. Note that we mainly consider appointments for the primary resources, i.e., the physicians. This simplification assumes that the non-physician tasks corresponding to the appointment/patient-physician contact are performed along the way. Also, note that there is a significant overlap between the appointment planning and capacity planning literature since both problems are often addressed simultaneously.

Recently, Ahmadi-Javid et al. [3] reviewed optimization studies in outpatient appointment systems in healthcare. Further reviews on appointment scheduling in healthcare are presented in Cayirli and Veral [14] and Gupta and Denton [37].

#### Access policy and admission control

On the tactical level, a medical practice decides on an access policy; see Table 4. In the traditional policy, every (non-urgent) patient needs to book an appointment ahead of time. In the advanced access or open access policy, patients can book appointments on the same day (or maybe the next day) only, and must be seen by the physician on that day. A hybrid or carve-out policy lets patients schedule appointments ahead of time while also reserving capacity for same-day demand. Scheduling appointments gives the physician some control over the demand and helps to reduce the uncertainty of incoming requests. However, if appointments are offered for future days, patients experience longer access times, and the practice is confronted with uncertainty due to cancellations and no-shows. Both Robinson and Chen [89] and Cho and Cattani [17] compare the traditional policy with the open access policy and conclude that the open access policy dominates the traditional policy in most considered settings.

Then, admission control decisions define to whom appointments are offered, for what, when, where, and with which physician or non-physician staff member to ensure access to care for all patient types. Also, they define who is allowed to walk in, for what reason, when, and to see which physician or non-physician staff; see Table 4. Dobson et al. [23] examine the effect of reserving slots for urgent patients on the number of rejected urgent patients and the queue length for regular patients with a stochastic model. Liu et al. [63] consider the capacity allocation to walk-in and scheduled patients as well, using a queueing model and considering that patients chose strategically between the two options.

#### Appointment scheduling

In appointment scheduling, we typically create session template schedules for physicians by assigning virtual patients to predefined appointment starting times or slots; see Table 5. Later on, actual patient requests are assigned to those reserved slots. Most of the literature on appointment scheduling considers minimization of overtime, idle time, and (direct) patient waiting time. Some papers integrate part of admission control and appointment scheduling by creating template schedules to minimize access time, also called indirect waiting time. Others even take into account both direct and indirect waiting times.

In a template schedule, we can differentiate between several patient classes with their individual parameters to minimize overtime, idle time, and patient waiting time. For example, we can consider cancellations, no-shows, walk-ins, patient time and physician preferences, unpunctual patients, stochastic service times, lateness, etc. Dantas et al. [20] present an extensive literature review on no-shows in appointment scheduling, showing that the main determinants of no-shows are high lead time and prior no-show history. Overbooking appointment slots and a maximal booking window may be used to counter the adverse effects of no-shows. For example, Leeftink et al. [58] use an analytical queuing model with time-dependent no-show and cancellation rates to determine the optimal booking horizon to minimize the effects of no-shows and cancellations and the cost of rejecting patients. Koeleman and Koole [52] computes appointment schedules, considering the arrival of emergencies, proposing a local search heuristic that can find the globally optimal schedule. Srinivas and Ravindran [97] propose the use of machine learning to classify patients with respect to their no-show probability. The authors use this classification and a service duration classification to propose appointment scheduling rules. Zacharias and Yunes [115] design appointment schedules considering no-shows, non-punctuality, general stochastic service times, and unscheduled emergency walk-ins. Kuiper et al. [54] calculate optimal appointment schedules considering no-shows and walk-ins and assuming phase-type distributed service times. There is also literature on appointment scheduling with multiple (identical) servers [53, 96, 114].

Schacht [92] aim to minimize access time using a stochastic mixed-integer linear program to determine an appointment scheduling template considering walk-ins and seasonality. Laan et al. [57] develop an appointment schedule using a stochastic mixed integer program considering time-varying demand and capacity.

Kuo et al. [56] consider direct and indirect waiting time together, using a scenario-based stochastic mixed-integer linear program to define a template schedule and an inter-session simulation model to handle the actual appointment booking. Cayirli et al. [15] investigate both how many slots to reserve for walk-in patients and scheduled patients, taking into account seasonal demand, and define appointment slots for scheduled arrivals using simulation.

#### Operational online appointment planning

On the (online) operational planning level, decisions are usually based on rules defined in previous higher-level decisions. However, we review some models that schedule actual patients online without previously defined template schedules. This relates to the patient-to-appointment assignment decision in Table 6. Feldman et al. [29] present a static and dynamic model to maximize the expected daily profit by deciding on a set of days to offer to patients for appointment booking. Patients have time preferences and may cancel or not show up for their appointment. Zander and Mohring [118] present a mixed-integer linear program to determine a set of appointment start times to offer to an appointment-requesting patient on a particular day, considering patient type-specific service times and time preferences. Liu et al. [62] consider non-sequential (one offer) and sequential (potentially several offers) appointment offerings to maximize the number of booked appointment slots where patients are assumed to have unknown time preferences. Zacharias et al. [116] present an analytical model to decide online on which day and at which time to schedule requesting (homogeneous) patients, taking direct and indirect waiting times into account.

Samorani and Ganguly [90] investigate the wait-preempt dilemma by building an analytical model that determines when a physician should wait to see a scheduled patient (who is late) or see an early patient right away, corresponding to the decision “Whom to serve next” in Table 6.

#### Data requirements

Historical data on demand and supply in appointment planning is mainly needed to predict future patient demand (potentially also dependent on supply decisions), considering the potential patient classification with respect to no-show probability, punctuality, etc. Often used input parameters to describe future demand are the distribution of daily requests, the service time distributions (possibly patient dependent), punctuality distributions (possibly patient dependent), cancellation and no-show probabilities (possibly patient, slot, or access time-dependent), walk-in probabilities (possibly slot dependent), and time and physician preferences of patients. To define those input parameters, we need historical demand data on the arrival of patient requests, booked appointments, cancellations, and patient arrivals in the practice (including walk-ins); see Section 3. Suppose we also want to model changes in patient demand based on changes in supply. In that case, we also need historical data on supply, such as offered services and available resources based on time and patient type combinations as described in Section 3.

Klute et al. [51] predict outpatient appointment requests using machine learning and traditional models, concluding that one should test various traditional, machine learning, and hybrid prediction methods to find the best one for a given data set.

### Access channels

Traditionally, patients meet their physician in person in the medical practice or sometimes during home visits. However, in recent years, telehealth, e-visits, and telephone consultations have become more common. Especially during COVID-19, these alternative access channels allowed contactless appointments to reduce risks to physicians and vulnerable patients. Considering the offering of different access channels can influence all the planning problems shown in Fig. 2. First, offering a certain access channel is a service design decision, as the access channel is an inherent part of a service, such as a consultation that happens online. As described in Table 2, the decision to offer certain services, and therefore also certain access channels, is based on the expected costs and revenues. Furthermore, deciding to offer certain access channels may influence strategic decisions such as capacity planning and layout planning, e.g., when a dedicated office for e-visits needs to be planned. It also influences (strategic) workforce planning and location planning, as, for example, physicians need to have the necessary skills to offer e-visits, and stable internet access (for physicians and patients) must be ensured. If home visits are offered, the physician must be able to drive.

Regarding tactical decisions, for example, when adding new patients to the panel, the expected demand for different access channels over time must be considered. In workforce planning, the access channels need to be part of the shift so that physicians with the corresponding skill sets will be rostered. In admission control, it needs to be decided which patient types are allowed to use which access channels, and in appointment scheduling, when a physician offers which types of access channels.

Access channels also play a role in operational decisions; for example, when a patient books or reschedules an appointment, the access channel needs to be decided on. The access channel may also influence the willingness to wait for a patient and, therefore, influence the decision of whom to serve next.

Several publications have addressed different aspects of providing and integrating e-visits into primary care. Bavafa et al. [8] study primary care delivery in the U.S. through on-site and e-visits and take a joint view on patient behavior and physician preferences. The authors analyse how offering e-visits impacts panel health, panel size, and physician earnings. Also for the U.S., Çakıcı and Mills [13] investigate effects of telehealth pay-parity and other payment alternatives with respect to allocating capacity to on-site and telehealth visits while taking the probability of duplicate visits into account. Zhong et al. [123] propose an analytical model based on queuing to characterize a primary care physician’s operations with e-visits and evaluate the performance. Queuing was also used by Prakash and Zhong [80] in an analytical framework for modeling the primary care delivery system to determine patient flows under different queue-joining behaviors. The aim was to enable an analysis of system configurations and their influence on system performance. Zhong [121] also apply queuing theory to model the appointment capacity problem for a primary care practice that also offers e-visits. Scheduling policies for on-site and e-visits in primary care practices have been analyzed by Zhong et al. [122]. The authors present analytical formulas to evaluate the mean and variance of the patient length of stay in the practice. Finally, there are dedicated websites to educate physicians about the use of telehealth in practice [10, 100].

## The best-case practice data set

Based on Section 2 and especially Fig. 1, we give an overview of relevant data to capture demand and supply that should be documented by a practice, if possible. Here, we structure the data by going over the involved people and objects separately. We start with data that represents demand, i.e., patient-practice interactions.

This is a list of data that is related to a specific patient and can be used for identification:

- Name/ID,
- Birth date,
- Gender,
- Address,
- Insurance types,
- Health status,
- Assigned physician, if any.

As this paper focuses on logistical data, we do not specify what will be documented in the health status, as this is outside of the scope of our expertise. We suppose it should include information on the patient’s condition, medication, blood work results, etc. If some of those patient attributes change over time, the changes and the time of the changes, together with the previous values, should be documented.

If a new patient requests to join a panel, a panel patient requests to change panels, or to leave the panel, we store the following data:

- Patient,
- Request day,
- Request time,
- Request mode (e.g., online, telephone, etc.),
- Corresponding appointment or walk-in event, if any,
- Physician(s) requested, if any,
- Rejection (yes/no) and reasons,
- balking (yes/no) and reasons,
- Assigned physician, if any.

Next, we list data that is related to an appointment request:

- Patient,
- Request day,
- Request time,
- Request mode,
- Urgency level,
- Times requested/offered (time of day, start time of appointment),
- Services requested/offered (e.g., first consultation, specific treatment, etc.; need to include the access channel),
- Resources requested/offered (e.g., physician, non-physician staff, equipment, room, etc.),
- Rejection (yes/no) and reasons,
- balking (yes/no) and reasons,
- Booked appointments, if any.

We now list data relevant to a booked appointment:

- Patient,
- Appointment request,
- Booking day,
- Booking time,
- Booking mode (e.g., online, telephone, etc.),
- Planned day,
- Planned start time,
- Planned duration,
- Planned services,
- Planned resources,
- Cancellation (yes/no),
- No-show (yes/ no) and reasons, if any.

Instead of using a start time and a duration, we can also register the booking of predefined time slots.

If the patient or practice cancels the appointment, we further consider:

- Cancellation day,
- Cancellation time,
- Cancellation reason,
- Cancellation unit (patient or practice),
- (Newly) booked appointment(s), if any.

Any change in a booked appointment can be documented as a cancellation together with the new appointment booked. This way, information on previous planning is saved.

If the patient shows up, we further consider:

- Arrival time,
- Reneging time, in case of reneging,
- Start time,
- Duration,
- Services,
- Resources.

If a patient walks in without an appointment, we store the following data for this event:

- Patient,
- Arrival day,
- Arrival time,
- Urgency level,
- Services requested/offered,
- Resources requested/offered,
- Rejection (yes/no) and reasons,
- Reneging time and reasons, in case of reneging,
- Booked appointments, if any, in case of rejection or reneging,
- Start time,
- Duration,
- Services,
- Resources.

In the following, we will present data related to describing supply. We need to connect the patient with services, resources, and a booking time to book appointments. Here, we start with data related to a service:

- Service name/ID,
- Access channel,
- Resource requirements,
- Time requirements.

Appointments are booked ahead of time. Therefore, we need data on the future (planned) availability of resources and time slots. The following data is resource-related:

- Resource name/ID (e.g., physician, non-physician staff, equipment, room, etc.),
- Time availabilities,
- Service capabilities,
- Non-patient-related work.

Any data changes before realization should be documented, storing the former and the new values together with the times those changes happened. After the realization, the actual working time of resources should be documented. Note that the working time of a resource may include work that cannot be assigned to a specific patient. This type of work could be planned as well. However, we do not consider this explicitly here.

For a combination of a resource and a time slot, we consider:

- Day of time slot,
- Start time of time slot,
- End time of time slot
- Resource,
- Booking modes (e.g., online appointment, walk-in slot, etc.),
- Patient types (e.g., panel patients, walk-ins, etc.),
- Urgency levels,
- Services,
- Start (and possibly end) time of booking availability, if applicable.

Here, a time slot is considered the greatest common factor of all planned service durations. It can be part of an appointment or a walk-in event. We do not explicitly define it here, but we can have further dependencies between time slots, booking modes, patient types, and services, e.g., a panel patient can only book an appointment via an online system. As explained before, resource and resource-time slot data should be expanded by a time component such that any changes can be retraced. This way, we know, for example, when a physician changed the time availability due to a planned vacation. The realization of a resource and time slot usage can be reconstructed using the data on realized appointments or walk-in sessions. Note that a time slot might not be used entirely to serve one patient by one resource in the realization.

If necessary, we can add another element, i.e., care pathways defined by a series of services. Then, considering the patient-practice interactions, we can replace services with care pathways.

## The importance of data collection

In this section, we briefly discuss the applicability of Operations Research models based on missing data concerning the best-case practice data set described in the previous Section 3. Note that the best-case practice data set includes all data points the practice has control over regarding collection. All the other data mentioned in Tables 1 to 6 (not related to historical demand and supply data) must be retrieved from other sources.

According to Table 1, historical patient demand data, specifically historical demand data for different practice types, is used in regional strategic planning to decide on the location and capacity of potentially new practices. In that case, the quality of decision-making by the regional authority depends on gathering individual practice data on patient demand volume. Similar data is needed for practice managers to decide on a location when opening a new practice; see Table 2. To solve the strategic problems of capacity planning, workforce planning, and layout planning, the practice manager would need even more detailed data on patient demand volume per service and care pathway as well as historical service durations; see Table 3.

For panel management, to plan demand and match it to capacity, we need to collect patient demand data per patient type over the years. Finally, most decisions based on historical demand data fall under appointment planning. To divide capacity among patient types (access policy and admission control in Table 4), we need to predict future requests, which is not possible if only booked and realized appointments are registered instead of appointment or walk-in requests. Fulfilling patient preferences in appointment booking can positively influence adverse effects such as cancellations, no-shows, walk-ins, and unpunctual patient visits. However, to predict future preferences, data on patient requests for specific time slots of physicians have to be registered. Further, the more details we collect about patients (e.g., no-shows, walk-ins, cancellations, service times, etc.), the better appointment schedules we can create. Note that the appointment scheduling literature cannot be applied if we do not gather data on realized service durations.

Note that higher-level decisions are usually more costly because they are fixed for longer time intervals or even permanent, such as location decisions. However, we also see that, especially for those higher-level decisions, the decision-makers usually do not have self-collected data. A practice first has to collect data over a considerable amount of time, until this data can be used for tactical and operational decision-making.

Historic supply data is beneficial to define input parameters for models that explicitly consider the influence of supply decisions on demand behavior, i.e., the flows along the arrows in Fig. 1.

Let us briefly review the possible monetary impact of incomplete data collection. If a GP in New Zealand fails to offer an appointment to a patient or underestimates the no-show rate and, therefore, ends up with an empty slot, the GP could easily lose 85 NZD for one 15-minute slot [108]. If this happens once a day and the practice is open for 245 days of the year, this adds up to a loss of 20,825 NZD in a year. Similar numbers could be derived for Germany [47] or the Netherlands, of course. Expected losses in the US, for example, might be even higher. In this context, it is important to note that not all the costs of missing or faulty data are that easy to estimate. For example, it is also important to take the quality of care and potential follow-up costs into account, e.g., in case a patient cannot get an appointment in time. Patient and staff satisfaction is important as well, and it is difficult to quantify this in relation to costs.

## Real-world data collection and decision-making

In this section, we take a closer look at data availability and decision-making for different healthcare systems, discussing this using the three countries of Germany, the Netherlands, and New Zealand as examples. The three countries differ in many aspects, including geographically, in terms of their sizes (area and number of inhabitants), and in the design of individual healthcare systems, including how they are funded. Nevertheless, they experience similar challenges regarding an increase in demand and cost pressure, as well as a staff shortage. In Germany, for example, there are not enough medical students wanting to pursue a career in general medicine, and those who do favor bigger cities and group practices that offer a better work-life balance [48]. Especially in Germany and New Zealand, providing access to ambulatory care in rural areas is challenging. While in Germany, a region is defined as rural if 140 inhabitants are living in one $km^2$, the average population density in New Zealand, for example, is only 18 inhabitants per $km^2$ [110], often leading to longer distances for patients to ambulatory care facilities and a less attractive environment for doctors to open a practice.

In Germany, the National Association of Statutory Health Insurance Physicians (Kassenärztliche Bundesvereinigung, KBV) is in charge of the needs- and demand-based planning and determines the number of doctors that can work in each region to care for publicly insured patients [49]. A license linked to a region is necessary to open up a practice. The Fifth Book of the Social Security Code (SGB V) [69] defines the legal regulations for demand-based planning.

In Germany and the Netherlands, patients usually pay for health insurance, which covers ambulatory care and GP visits. In New Zealand, hospital-based healthcare is covered by taxes, but patients need to pay for themselves to receive primary care. When patients enrol in a GP practice free of charge, the government will subsidize care, and patients will only pay a reduced consultation fee. Practices are private businesses that can set their own fees for consultations and other health services. While the fees charged must be within a certain threshold agreed to by the District Health Board and PHOs, practices determine the level of co-payment. Therefore, there are significant differences in fees between practices [68], which again can influence the demand and, consequently, capacity planning as well as panel management. In the Netherlands, the national government is responsible for regulating health insurers as well as (ambulatory) care providers [95].

In Germany and New Zealand, inhabitants can freely choose between medical practices, while in the Netherlands, you must register with a practice if you want to be seen by a doctor. In Germany and New Zealand, you are incentivized to do so. However, practices in these three countries do not necessarily need to accept new patients. In New Zealand, if a GP cannot take a patient, the practice refers the patient to their primary health organization (PHO) for help finding another practice. Unfortunately, this fact makes it more challenging to predict the demand streams, for example, when opening a new practice. Therefore, it influences the location and capacity planning problems together with panel management. In the Netherlands, primary care practices act as gatekeepers, and patients can only see a specialist with a referral from a GP [95]. In Germany, GPs also often still own their practices, and the fees per patient are fixed. Up until today, the budget for a practice has been capped. In New Zealand, GPs have a stronger incentive to offer better services or serve more clients in order to increase their income, leading, for example, to different settings in panel management or appointment planning.

In Germany, data collection is fairly difficult, especially if decisions on the regional strategic level are to be made. There is no one stakeholder in Germany that has all the relevant data, and it would need to be collected from each practice individually. Data availability and quality can differ significantly between the practices. When making decisions for a new practice, doctors and/or practice owners have only limited access to data, maybe from their predecessors if they are taking over an already existing practice, but no other patient or demand data from the region. While in New Zealand, data access for research is not trivial as well, it is possible to pay a provider to retrieve all the necessary data with respect to one practice, as well as on a regional (or even national) level. As patients usually register with a GP practice, information about that also exists and can potentially be accessed, e.g., in order to make better assumptions on how patients choose a practice within (regional) location planning models and demand predictions. Due to GP practices acting as gatekeepers to the healthcare system, data collection is more structured than in Germany.

### Real-world appointment data sets

We were able to receive four (appointment) data sets from group practices of general practitioners (GP), urologists (U), cardiologists (C), and otolaryngologists (O) in Germany. The urology and cardiology practices use the same administration software. For three out of four data sets, a row describes an appointment. Sometimes, those appointments can be walk-ins, but this can only be identified by comparing the booking time and the planned start time of the appointment. A row in the data set from the general practitioner practice relates to a charged service for the patient. A subset of those rows relates to an actual patient-physician contact. In Table 7, we show the available appointment data attributes for each data set. These attributes relate to attributes used in Section 3 for booked appointments, cancellations, and patients who showed up or walked in. In the table, “$\checkmark$” stands for available,“($\checkmark$)” stands for partly available, and “–” for not available.

**Table 7:** Available information for booked appointments in the four data sets

Data points	GP	U	C	O
Patient ID	$\checkmark$	$\checkmark$	($\checkmark$)	$\checkmark$
Booking day and time	–	$\checkmark$	$\checkmark$	$\checkmark$
Booking mode	–	–	–	$\checkmark$
Planned day and start time	$\checkmark$	$\checkmark$	$\checkmark$	$\checkmark$
Planned duration	–	$\checkmark$	$\checkmark$	$\checkmark$
Cancelation day and time	–	($\checkmark$)	($\checkmark$)	–
No-show	–	$\checkmark$	$\checkmark$	$\checkmark$
Services	$\checkmark$	$\checkmark$	$\checkmark$	$\checkmark$
Resources	$\checkmark$	$\checkmark$	$\checkmark$	$\checkmark$

We first notice that the most critical information, i.e., the information of the planned day and start time of the appointment, the corresponding patient, services, and resources, is available in all four data sets. This information is necessary for accounting, i.e., to receive payment from the insurance companies. However, the cardiology practice does not use unique patient IDs when storing appointment data, making it challenging to recognize revisiting patients. Three out of four practices document the day and time of the booking and the duration of the planned appointment. The booking mode is only registered by the otolaryngology practice. In all four practices, if an appointment is cancelled, the corresponding data row is deleted or overwritten to contain values for a new appointment. However, the urology and cardiology practices include a column that shows the date and time when the appointment was last changed (the corresponding previous values are lost). Surprisingly, no-shows are only documented in three of the four practices. Another problem is that planned data entries are overwritten by the realized values concerning services and resources.

No information is stored on requested or offered appointment start times, services, or resources. Further, information on appointment requests that do not lead to an appointment booking (balking or rejection) is lost. There is no knowledge stored of why appointments were cancelled, by whom, or if a new appointment was booked. Considering the realization of the planned appointment, no data is collected besides the provided services and the corresponding resources. Therefore, no data is stored on patient arrival times, reneging patients (who will probably be registered as no-shows), or service durations. The data sets also do not explicitly consider any information on panel requests or the time availabilities and service capabilities of resources. Some of this data will probably be available in those practices, but not as part of the appointment data collection.

Based on the tables in Section 2 and the findings from Section 4, we describe the consequences in terms of decision-making for those four practices. Here, we mainly consider tactical and operational decisions since strategic decisions have already been made. For all four practices, their data collection does not enable them to establish the original demand, including panel requests, rejections, balking, reneging, and preferences. Therefore, decision-making concerning panel management, access policies, and admission control will be hard.

It can not be determined if a slot stayed idle due to a late cancellation or low demand. Since late cancellations have similar adverse effects as no-shows, we may underestimate the effective no-show rate. Further, no information on actual service durations, patient lateness, or reneging is available. Therefore, crucial input parameters are missing for appointment scheduling.

## Barriers to applying operation research models in practice

There is vast OR literature on planning and control decisions for outpatient clinics. However, few reports show the actual application of their methods in practice. There are numerous reasons why methods are not applied. In this section, we comment on the potential reasons for barriers to applying Operations Research models in practice, mainly connected to data.

We already saw in Sections 4 and 5 that data availability is a big problem. Especially for strategic decisions at the regional and practice levels, there is usually no self-collected data available, and decision-makers must rely on other data sources. Hence, decision-making can be improved by making historical demand and supply data from various practices available. Further, practices should start collecting data strategically by making the connection to the decision problems that are most important to them, e.g., through the usage of our tables.

Besides numerous other requirements, one condition must be fulfilled to apply any method to solve a planning and control problem: We need to define the model parameters. To this end, we first need (historical) data and then instructions on deriving the model parameters from this data, which is not always straightforward. For example, defining an appointment request rate based on booked or realized appointments may underestimate the actual rate and lead to poor decisions. Ideally, modelers should put a stronger emphasis on making an impact with their work and think more about how practitioners can use their models. They should indicate the minimal data requirements of their models and deliver instructions on how to derive the model input parameters from data. In case of available relevant real-world data of sufficient quality, the parameter definition process should be illustrated together with the model’s description. However, suppose they cannot illustrate this process with real-world data. In that case, we recommend that modellers describe the data that should be collected and explain the derivation of the potential parameters to prove that their model can be applied in practice. This can be done using the best-case data set from Section 3.

Another problem might be the proposed models in the literature themselves. One reason could be that they do not capture the complex reality enough. For example, most models do not consider the influence of supply decisions on demand behavior. Another reason for missing model applications in practice is that the objectives of the practitioners and the objective functions used by modelers are not aligned. For example, Kuiper et al. [55] conduct interviews with ten outpatient clinics. They find that the appointment slot length used in those clinics is never based on data. The trade-off between idle and waiting time, which is widely used in theory, is not recognized or applied. On the contrary, clinicians see idle time as a minor issue because they can switch to other work. Still, in general, the clinics use tight schedules. Hence, close collaboration between modelers and practitioners is recommended to ensure the alignment of objectives.

From a broader perspective, physicians and nurses who work in ambulatory care mostly do not have any experience or knowledge about OR/MS and hardly any time to get into it. With a multitude of practices in the countries, educating them is not trivial. It would help if software companies offered planning problems integrated into tools to use in practice, but due to the different healthcare systems, country-specific solutions might be necessary, leading to high investments, which companies might not be willing to make without financial support or guaranteed customers paying for the solutions. Again, the high number of often small practices poses a significant challenge. Those barriers to implementing OR/MS solutions in practice do not only exist for ambulatory care but have been studied for other healthcare areas like emergency services [87].

## Conclusion and outlook

In this paper, we review the planning problems and corresponding planning and control decisions that need to be made by a practice manager when opening and running a medical practice, and connect those to the required data. We give an overview of demand and supply data that practices can document and comment on the consequences for decision-making when specific data is not collected. We consider the influence of healthcare systems on data collection and decision-making, as well as real-world appointment data sets. Finally, we discuss barriers to applying Operations Research models in practice. To eliminate those barriers, we must educate practice managers and software companies about data collection and the connection to planning problems and decisions. We, as researchers, need to work closely with practice and put more emphasis on building models tailored to specific practice settings with their potentially available data and explaining how to derive the model input parameters from this data. Further, to overcome the problem of data availability when opening a new practice, we suggest building accessible meta-collections of several data sets from real-world practices.

Based on our insights, we propose to further study the connection between decisions and data requirements in ambulatory care logistics. We believe that more realistic and powerful Operations Research models can be created by explicitly considering the influence of supply decisions on patient demand. For example, supply decisions influence access times that can, in turn, influence appointment request rates, no-show probability, cancellation probability, walk-in probability, etc. It would further be interesting to investigate the effect of the fulfilment or non-fulfilment of patient preferences concerning panel or appointment requests on the cancellation and no-show probability.

In general, modelers should continue to integrate as many decisions as possible. For example, panel management and staffing adjustments could be reasonably solved together. Further, more integration would allow us to create models to make online decisions on lower-level decisions, such as the acceptance of appointments or walk-in requests, without having to follow the rules set on higher levels. However, existing models can be extended by considering more patient characteristics, such as no-show or lateness probabilities that depend on the day or the time.

Further, we suggest future research to build a taxonomy of different practice settings, including a list of relevant data indicating their relative importance. In this context, accessible meta-collections of several data sets from real-world practices would be hugely beneficial for regional decision-makers as well as practice managers who want to open a new practice, especially for costly strategic decisions. However, when setting up such a data collection, potential ethical, privacy, and legal issues, such as data anonymization, have to be addressed first.

## Data Availability

The data used in the manuscript is confidential and will not be deposited.
